# Droplet centrifugation, droplet DNA extraction, and rapid droplet thermocycling for simpler and faster PCR assay using wire-guided manipulations

**DOI:** 10.1186/1754-1611-6-15

**Published:** 2012-09-04

**Authors:** David J You, Jeong-Yeol Yoon

**Affiliations:** 1 Department of Agricultural and Biosystems Engineering, The University of Arizona, Tucson, AZ 85721-0038, USA

**Keywords:** Droplet manipulations, *Escherichia coli*, Peptidase D, Droplet PCR, Rapid PCR

## Abstract

A computer numerical control (CNC) apparatus was used to perform droplet centrifugation, droplet DNA extraction, and rapid droplet thermocycling on a single superhydrophobic surface and a multi-chambered PCB heater. Droplets were manipulated using “wire-guided” method (a pipette tip was used in this study). This methodology can be easily adapted to existing commercial robotic pipetting system, while demonstrated added capabilities such as vibrational mixing, high-speed centrifuging of droplets, simple DNA extraction utilizing the hydrophobicity difference between the tip and the superhydrophobic surface, and rapid thermocycling with a moving droplet, all with wire-guided droplet manipulations on a superhydrophobic surface and a multi-chambered PCB heater (i.e., not on a 96-well plate). Serial dilutions were demonstrated for diluting sample matrix. Centrifuging was demonstrated by rotating a 10 μL droplet at 2300 round per minute, concentrating *E. coli* by more than 3-fold within 3 min. DNA extraction was demonstrated from *E. coli* sample utilizing the disposable pipette tip to cleverly attract the extracted DNA from the droplet residing on a superhydrophobic surface, which took less than 10 min. Following extraction, the 1500 bp sequence of Peptidase D from *E. coli* was amplified using rapid droplet thermocycling, which took 10 min for 30 cycles. The total assay time was 23 min, including droplet centrifugation, droplet DNA extraction and rapid droplet thermocycling. Evaporation from of 10 μL droplets was not significant during these procedures, since the longest time exposure to air and the vibrations was less than 5 min (during DNA extraction). The results of these sequentially executed processes were analyzed using gel electrophoresis. Thus, this work demonstrates the adaptability of the system to replace many common laboratory tasks on a single platform (through re-programmability), in rapid succession (using droplets), and with a high level of accuracy and automation.

## Background

Manipulating small droplets for analytical methods has been the focus of much attention in recent years. The use of small droplets allows for significantly lower reaction volumes and decreased assay times for many common laboratory procedures. Droplets also allow for sample isolation in a small format, allowing for multiple sample analysis through discrete, independent manipulation and quantification. Furthermore, it has been established that complex and reconfigurable bioanalysis and biorecognition is only possible with droplets [1]. The two primary modes of droplet manipulations are: (1) to use discrete liquid plugs in pre-defined microchannels [2,3], or (2) to use droplets sitting on an open, flat surface [4,5]. Although the former (liquid-plug type) has been popular in digital microfluidics, the latter (open-surface type) has more potential as its reaction protocol can be reprogrammed to whatever combination one can conceive. Furthermore, while complex and reconfigurable algorithms can be implemented into pre-defined microchannel systems through the use of elastomeric microvalves [6,7], these microfluidic large-scale integrations (mLSI) systems typically require an array of external pneumatic solenoid valves, access to compressed air or a portable air compressor, and a control system with computer in order to choreograph precise, controlled droplet manipulations, requiring the addition of more components based on the complexity of the pre-defined microchannel layout. The manipulation of droplets of an open, flat surface has been demonstrated most notably with magnetofluidics. In magnetofluidics, droplets containing paramagnetic particles move over a superhydrophobic surface under the influence of an external magnetic field [8]. However, paramagnetic particles need to be designed as not to interfere with biological reactions, a capability that has not yet been confirmed fully. Another common technique is electrowetting-on-dielectrics (EWOD), which allows for precise droplet movement, splitting, and merging [9]. However, this method is comparatively more difficult to fabricate and operate, and has limitations with diffusional mixing and contamination from increased wetting on the surface [10]. Wire-guided droplet manipulations offer a simpler method for manipulating droplets on an open surface. Although a clean, metal wire was initially used to guide a droplet on a superhydrophobic surface (deriving the term *wire-guided*) [11], the wire can be replaced with a variety of materials and sizes to modulate the friction force of the droplet to the wire (the work of adhesion, *W*_*a*_), making the system highly adaptable to a wide range of droplet volumes and properties. Furthermore, the use of a syringe needle or disposable pipette tips can be used to perform precise droplet splitting and mixing with an attached vibration motor to the linear actuated syringe plunger. Using the wire-guided system to form pendant droplets on the ends of syringe needles has also been demonstrated for rapid PCR thermocycling, by using forced convective heat transfer through movement of the droplet in submerged silicone oil [12].

This work demonstrates the use of wire-guided droplet manipulations to perform a series of laboratory tasks on a single superhydrophobic surface measuring 25 mm × 55 mm and a multi-chambered PCB heater. The first step includes the execution of a series of 10-fold serial dilutions of a cultured *Escherichia coli* sample. Following dilution, the sample is then concentrated through a novel method of centrifugation by spinning the droplet around a metal syringe needle at a high rate by using a vibration motor under pulse width modulated (PWM) control. During centrifugation, the diluted sample at the center of the axis of rotation is drawn into the syringe, leaving a concentrated sample for further analysis. The sample then undergoes rapid DNA extraction, in which wire-guiding offers a clever approach to extracting and separating the precipitated genetic material. And afterwards, a genetic sequence is amplified through rapid thermocycling using a moving, pendant droplet, and the results confirmed by gel electrophoresis. In summary, we are presenting a reprogrammable, reusable, and reliable wire-guided droplet manipulation system that can concentrate bacterial samples, extract DNA, and perform rapid droplet thermocycling in microliter scale capable for subsequent DNA analysis. We demonstrate these droplet manipulation processes by using disposable pipette tips. Thus our methodology can be effortlessly adopted with ease by commercially available robotic pipetting systems, while demonstrated added capabilities such as vibrational mixing, high-speed centrifuging of droplets, simple DNA extraction utilizing the hydrophobicity difference, and rapid thermocycling with a moving droplet.

## Results and discussion

### Droplet serial dilution and droplet centrifugation

**Figure 1:**
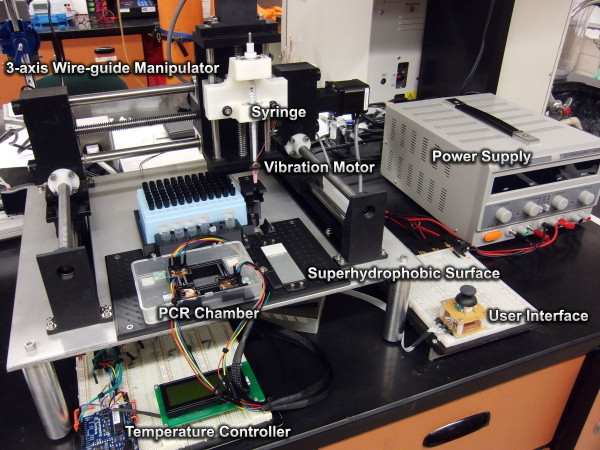
**Wire-guided droplet manipulator apparatus.** Modular design includes PCR chamber for rapid-PCR thermocycling and a superhydrophobic surface for serial dilution, centrifugation, and DNA extraction.

To demonstrate precise sample handling in a reconfigurable manner, serial dilutions were performed to simulate a diluted bacterial sample. This diluted sample was then re-concentrated using wire-guided droplet centrifugation, which only the target is concentrated while culture media (and potentially other sample matrices) is diluted, thus effectively eliminating much of culture media or sample matrices. The genetic material was extracted using the same droplet manipulation assay surface with only a different pre-programmed algorithm, demonstrating the ease of reconfigurability of the system. Afterwards, the DNA was amplified using rapid droplet thermocycling with wire-guided droplet manipulation. Figure 1 shows the entire apparatus to perform all the aforementioned protocols. The modular base allows for easy repositioning and addition of further components to the system. While only a single confluent assay was demonstrated, the ability to run multiple assays simultaneously, utilizing the necessary delays between steps in the protocols, could easily be implemented.

**Figure 2:**
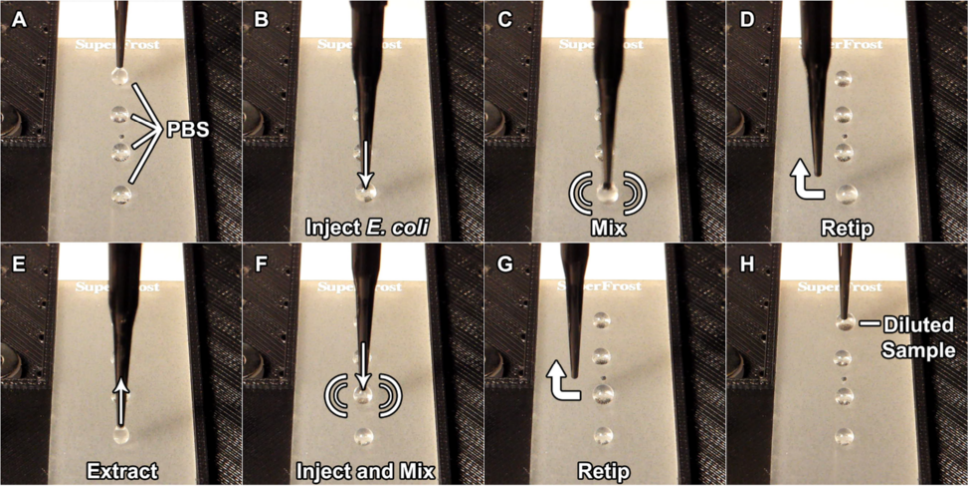
**Serial dilution protocol for *E. coli *sample A-H).** Wire-guided syringe needle manipulates, splits, and mixes sample on a hydrophobic surface. Mixing performed with a vibration motor.

Figure 2 (and Additional file 1) illustrates a representative algorithm of the serial dilution protocol, in which an initial 20 μL droplet of PBS was mixed with 2 μL of *E. coli* cultured in LB broth, and serially diluted 4 times. Standard plate counting revealed consistent dilutions attributed to the accuracy of the linear actuator in conjunction with the 1 mL disposable syringe needles. Clearing the surface for the next set of procedures was as simple as either extracting the droplets and then dumping the pipette tip, or simply guiding the droplet off of the surface into a KimWipes to be absorbed and later discarded.

**Figure 3:**
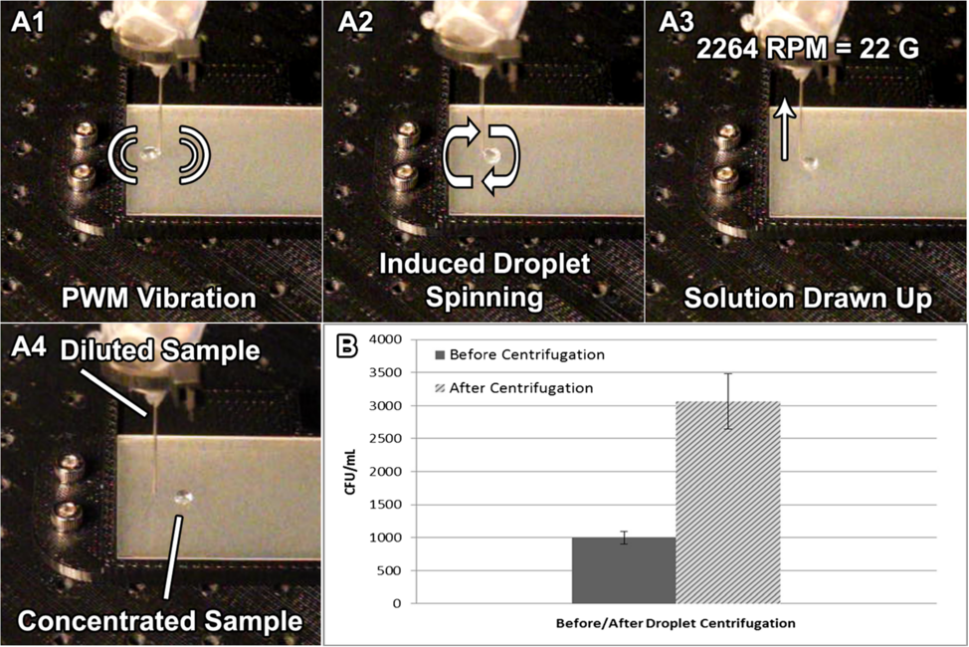
**Concentration of *E. coli *sample through droplet centrifugation A1**-**A4**). Centrifugation snapshots. Pulse-width-modulated (PWM) control of vibration motor induces circular motion path of droplet. **B)** Concentration of *E. coli* in 10 μL sample before and after droplet centrifugation revealing a 3.06 fold increase in mean concentration after 3 min.

To increase the concentration of the target bacteria for improved PCR detection, wire-guided droplet centrifugation was performed for a 10 μL droplet through the use of a long, 22-gauge stainless steel blunt-ended needle upon which a vibration motor was securely attached (Figure 3 and Additional file 1). Careful consideration was used in the positioning of the vibration motor to generate the best circular motion path, as verified by high-speed imaging. To maximize the number of rotations per minute (RPM), a pulse-width-modulation (PWM) algorithm was used to isolate a resonant frequency that induced the most stable and rapid circular motion path. This frequency was found to change between samples due to variations in needle height from the surface, contact angle to the superhydrophobic surface (typically 155 ± 2°) due to variations in droplet content, and the connection between the needle and syringe. Therefore, small changes to the PWM frequency was made through manual adjustment via the user interface. In practical applications, it would be beneficial to provide a set of such PWM frequencies depending on the type and size of the sample and needle to eliminate the need for manual adjustment.

After a specific period of centrifugation, the syringe automatically extracted the solution from the droplet. This extraction occurred at a much slower rate (~0.5 μL s^-1^) to reduce potential turbulence induced by the extraction process and subsequent decrease in droplet diameter. The concentration of *E. coli* before centrifugation and in the final droplet was analyzed using standard plate counting methods. Figure 3 B reveals the concentration of *E. coli* in a 10 μL sample before and after droplet centrifugation revealing a 3.06 fold increase in the mean concentration after only 3 mins.

**Figure 4:**
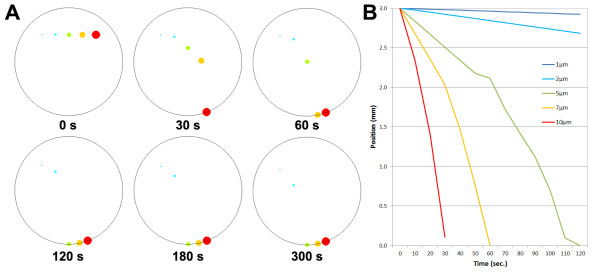
**FLUENT® simulations with 1, 2, 5, 7 and 10 μm aggregated *E. coli *particles (specific gravity = 1.2) in a 10 μL droplet under the effects of droplet centrifugation with a maximum RCF (relative centrifugal force) of 22.**

FLUENT® simulations were employed (Figure 4) to analyze the predictive movement of *E. coli* particles using single colony size (1 μm diameter = size of a single *E. coli*) and multi-cell colony sizes (2, 5, 7 and 10 μm diameter) and their location from the bottom versus time. When the diameter of the *E. coli* colony was 5, 7 and 10 μm, it took 120, 60 and 30 s respectively to settle down to the bottom of the droplet. For the cases of 1 and 2 μm, the particle never reached the bottom even after 300 s, however they did move by ~100 μm and ~300 μm, respectively, suggesting that this method is capable of centrifuging single *E. coli* colonies after only a short period of time. The simulation results indicate that longer periods of centrifugation for single colonies may be sufficient for complete centrifugation.

### Droplet DNA extraction and rapid droplet thermocycling

**Figure 5:**
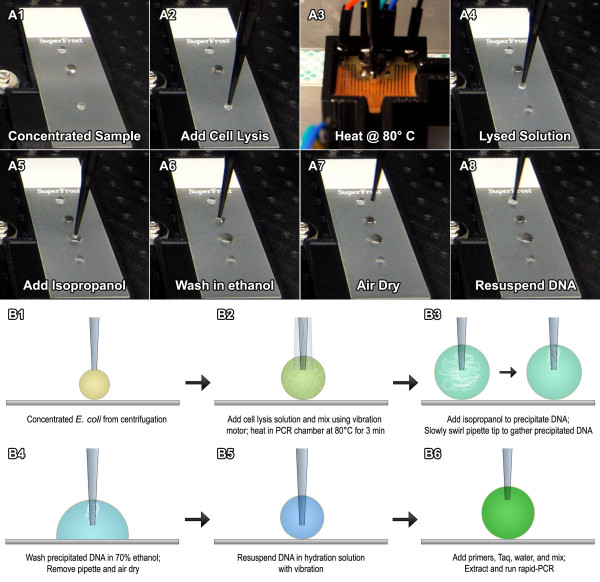
**A1-A8) Rapid DNA extraction of concentrated sample.A1**-**A8**) DNA extraction snapshots. **B1**) Concentrated sample of *E. coli* from droplet centrifugation step. **B2**) Nuclei lysis solution is added, mixed, and heated in lysis chamber at 80°C for 5 min. **B3**) Isopropanol (IPA) is added to lysed sample to precipitate DNA, which adheres to the pipette tip. **B4**) After drying from isopropanol, the DNA-saturated pipette tip is washed in ethanol and dried. **B5**) The DNA is resuspended in hydration solution. **B6**) The DNA solution is prepared for rapid-PCR thermocycling.

The extraction and amplification of genetic material from *E. coli* was performed using the same apparatus configuration and on the same superhydrophobic surface. The only difference is the execution of a different pre-programmed algorithm. Figure 5 (and Additional file 1) illustrates a representative algorithm of the DNA extraction protocol, in which the concentrated droplet of *E. coli* from the previous centrifugation step undergoes a series of lysing, precipitation, washing, and rehydration steps to prepare the genetic material for amplification and subsequent detection. However, our method of droplet DNA extraction cannot effectively remove the culture media, therefore, we evaluated the performance of our amplification with and without sample media present, both with our system and a commercial benchtop thermocycler.

Figure 5 B presents a 6 step protocol for the extraction of DNA from *E. coli*. An essential component of this protocol is step 3, in which the precipitated DNA/proteins following cell lysis is cleverly extracted from the droplet using the pipette tip as the substrate. Because of the hydrophobic effect of isopropanol precipitation, there exists an attraction of the genetic material towards the polymer pipette tip during removal, making the extraction process fast and easy, with minimal residual fluid needing to be evaporated. This same procedure was initially attempted in a 2 mL centrifuge tube, but when the precipitated material left the fluid and entered the air at the empty upper portion of the tube, the absence of a polar medium caused the material to immediately attach to the inside of the tube, making extraction difficult and inconsistent. Furthermore, rehydration of the extracted DNA in rehydration buffer from the pipette tip utilizing the vibration motor required only 3 min. This process could be further sped up by using heat to reduce the rehydration time to less than a minute, making the entire DNA extraction process from start to finish in less than 10 min.

**Figure 6:**
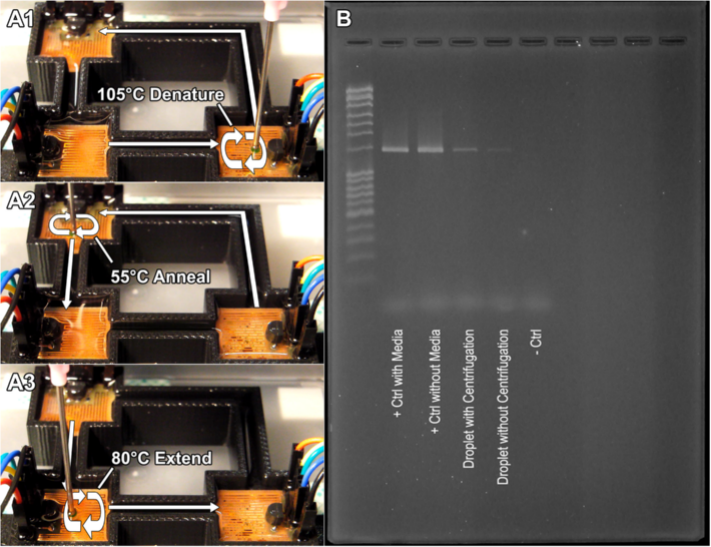
**Rapid droplet thermocycling.A1**-**A3**) Rapid droplet thermocycler executes 30 cycles in 10 min with ~1500 bp sequence through forced convective heat transfer under silicone oil submersion. **B**) Gel electrophoresis results for sample undergoing droplet centrifugation, DNA extraction, amplification, and rapid-PCR, all with wire-guided droplet manipulations. Lane 1 shows positive control of DNA extracted with culture media and lane 2 shows positive control of DNA extracted without culture media. Since culture media does not interfere with DNA extraction, lane 3 shows droplet with centrifugation and lane 4 shows droplet without centrifugation, demonstrating an increase in signal intensity of the PCR band through effective sample concentration by wire-guided droplet centrifugation.

Immediately following extraction, the droplet containing the genetic material underwent a 30-cycle thermocycling process using the rapid droplet PCR method that was previously established [11] (Figure 6 A). We used relatively long genetic sequence (~1500 bp) to make sure the rapid thermocycling could be applied to more complicated (thus more specific) target sequence. Because of this, the algorithm required a slightly longer extension time, requiring 10 min overall to perform 30 cycles. Previous work demonstrated PCR amplification of a 160 bp sequence in 6 min 50 s for 30 cycles. Figure 6 B is a gel image of the final results for the amplified ~1500 bp genetic sequence of *Escherichia coli*. Lane 1 is the ladder and confirms the ~1500 bp sequence being amplified. Lane 2 is the positive control with culture media, and lane 3 is the positive control without culture media. Pelletizing the *E. coli* with droplet centrifugation is not possible, and therefore the culture media could not be completely removed using this methodology; therefore the extent of inhibition of the DNA amplification was qualitatively determined by the positive controls in lane 2 and 3, showing that there is a minimal reduction in band intensity, resulting from a lower concentration of the amplified sequence, however, the strong positive band of lane 2 suggests that droplet PCR amplification in the presence of culture media remains a viable option.

Lane 4 is the result of the wire-guided droplet dilution, centrifugation, extraction, and amplification of *Escherichia coli* cultured in LB media. Lane 5 is the result using the identical processes of lane 4, minus the concentrating step from droplet centrifugation. The band intensity of lane 4 is much stronger than that of lane 5, further lending to the process of droplet centrifugation as a useful method for concentrating samples in the interest of either increasing the positive signal of PCR amplification or to reduce the number of cycles required for a positive result, decreasing assay time.

### Overall performance and evaporation issue

The total assay time is 23 min, including droplet centrifugation, droplet DNA extraction and rapid droplet thermocycling (i.e., minus the time for gel electrophoresis, which can be replaced with real-time quantification).

The potential evaporation of the reagents on the superhydrophobic surface was taken into consideration during these procedures. Steps in the protocols with the greatest time exposure to air and the vibrations, such as the rehydration of DNA into buffer (3 min with vibrations) and the droplet centrifugation (3 min with rapid rotations), did not show any issues with evaporation in our study. In fact, many contact angle measurements are made using 10 μL droplets within 10 min time frame, with no apparent evaporation issue. However, the use of smaller droplets (<1 μL) may be more susceptible to evaporation, especially during the centrifugation and vibrational mixing steps. Further work includes the manipulation of droplets completely submerged in silicone oil.

## Conclusions

This work demonstrates the use of a CNC apparatus to execute pre-programmed droplet movements and manipulations on a superhydrophobic surface measuring 25 mm by 55 mm and a multi-chambered PCB heater for the rapid detection of *E. coli* from PCR detection. By using a wire (here a pipette tip) to guide droplets along a superhydrophobic surface, there are no limitations on the complexity and configuration of procedures that it can perform, making it extremely versatile and far-reaching in its applications. The only modification required for adding or implementing changes for a new protocol is through simple pre-defined programming, which can be easily accomplished by any user with minimal programming knowledge. Because of this characteristic, the user has the ability to start and stop the assay(s) at any time, manually control the droplet system with a computer, make adjustments to the protocol on-the-fly, and resume where it left off. Alternatively, multiple protocols can be pre-loaded within the device and non-expert users can choose the appropriate protocol depending on the nature of sample. This methodology can be easily adapted to existing commercial robotic pipetting systems, which can further increase throughput with multi-tip systems.

Thus, this technology will potentially have a substantial impact in areas where the types of samples being tested may be unforeseen, and thus the protocols need to be adjusted (or chosen from the pre-loaded set of protocols) on the fly in the field, such as in food safety, forensics, or medical diagnostics. Furthermore, small changes in the protocols or reagent concentrations are common practice for optimizing PCR results, and this system is easily capable of such changes, giving it a huge advantage over other pre-determined microfluidic PCR platforms.

The system used in this work represents a benchtop apparatus that is highly adaptable to other benchtop pipetting system due to the simplicity of wire-guided droplet manipulation. Because the method employed is not highly-complex, it alludes to the ability for further miniaturization, making a potentially portable device for performing a nearly limitless number of protocols, including all of their variations. Future works includes the development of a handheld device for use in the field using an encapsulated silicone oil bath system cartridge. A one-time use cartridge makes contamination a non-issue, and the closed system prevents messy operation and ensures droplet stability while submerged. The design includes a circular chamber, as opposed to an orthogonal chamber, reducing the number of step motors to just two for complete operation. The encapsulated cartridge, circular design and reduced number of step motors will make the final device substantially smaller. Thus, this work demonstrates the reconfigurability of the system to replace many common laboratory tasks on a single platform (through reprogrammability), in rapid succession (using droplets), and with a high level of accuracy, leading towards an all-in-one, portable assay system.

## Materials and methods

### Computer-controlled wire-guided manipulator

The computer-controlled wire-guided apparatus was fabricated and assembled for manipulating droplets on both the superhydrophobic surface (for serial dilution and DNA extraction) and in the silicone oil-filled PCR chambers for thermocycling. The superhydrophobic surfaces made from nanocoatings of fluoropolymer on standard glass microscope slides were purchased from Surface Innovations (Durham, England). These nanocoatings create air pockets and put a droplet in metastable Fakir state. The base was constructed of aluminum onto which Thomson 12.7 mm linear rails and twin ball bearing pillow blocks (Thomson Industries, Radford, VA, USA) were mounted, allowing precise movement along the X, Y, and Z axis. Computer-controlled linear movement of each axis was performed with Nema 17 stepper motors obtained from Automation Direct (Cumming, GA, USA), connected to 12.7-10 mm Delrin® lead screw assemblies obtained from Precision-CNC-Router (Melbourne, FL, USA). Stepping of each motor was performed with an Arduino Mega microcontroller and Easydriver stepper motor controllers utilizing A3967 chips (SparkFun Electronics, Boulder, CO, USA). The stepper motors were powered by a 0–30 V, 3 A bench-top power supply (Marlin P. Jones and Associates, Inc., Lake Park, FL, USA). All of the mechanical components of the system were designed using SolidWorks 2010 (SolidWorks Corp., Concord, MA, USA) and then stereolithographically printed using a Dimension 1200ES 3D printer (Stratasys, Inc., Eden Prairie, MN, USA) in an acrylonitrile butadiene styrene (ABS) polymer.

For droplet insertion and extraction, disposable syringes were used with disposable pipet tips or modified Luer-Lok blunt-ended needles. 1 mL disposable plastic syringes with Luer-Lok tips were obtained from Fischer Scientific, and fitted into a quick-release plastic holder attached to the Z-axis of the apparatus. Precise droplet insertion, extraction, and manipulation was performed with either Beckman Span-8 black pipet tips (Beckman Coulter, Brea, CA, USA) or 14 gauge (1.628 mm inner diameter) blunt-ended needle (OK International, Garden Grove, CA, USA) and automated with a 5 V mini linear step motor (Marlin P. Jones and Associates, Inc.) attached to the syringe plunger.

Periodic calibration of the system was performed using a series of infrared photo interrupters (SparkFun Electronics) integrated into the X and Y planes. Black pipet tips were used as an opaque medium to trigger the photo interrupters. Calibration of the Z plane occurred during removal of the pipet tip.

### Droplet serial dilution

Serial dilution of a concentrated sample of *E. coli* culture was performed to demonstrate the application of the wire-guided droplet manipulation apparatus towards rapid and automated droplet serial dilution and subsequent vibrational mixing on a superhydrophobic surface. 20 μL droplets of PBS were deposited onto the surface as diluent. Following diluent deposition, 2 μL of *E. coli* culture were added to the first droplet and mixed using the attached vibration motor. The pipet was then discarded, during which the system simultaneously recalibrated itself to ensure device precision, and a new one obtained automatically from the replaceable pipet box. 2 μL of the first dilution was then extracted and deposited into the second droplet and mixed. This process was continued 4 times during the demonstration, creating a 10^-4^ dilution from the original *E. coli* culture. The precision of the system to perform serial dilutions in droplet platform were evaluated using standard plate counting methods for *E. coli*.

### Droplet centrifugation

Concentration of the diluted sample was accomplished through the use of droplet centrifugation. The apparatus consisted of a 22-gauge blunt-ended syringe needle (OK International) attached to a vibration motor (SparkFun Electronics) and controlled using pulse-width-modulation (PWM) via the Arduino Mega microcontroller. The droplet was centrifuged for 5 min at which point the solution sample from the center of the droplet was extracted, leaving a concentrated sample. Standard colony plate counting methods were used to determine the concentration of *E. coli* in the sample before and after centrifugation. Furthermore, to determine the effect of evaporation towards sample concentration (not centrifugation), an extracted DNA sample solution was used and three case studies were compared by measuring the DNA concentration with a UV–vis spectrophotometer (Nanodrop 2000; Thermo Scientific, Wilmington, DE, USA): 1) stock concentration, 2) concentration after leaving droplet stationary on superhydrophobic surface for 5 min, 3) concentration after spinning droplet for 5 min using wire-guided droplet centrifugation.

A Casio Exilim EX-FH100 (Casio America, Inc., Dover, NJ, USA) was used to acquire high speed video capture of the droplet rotation about the syringe needle at 480 frames per second.

### Droplet DNA extraction

Following concentration of the *E. coli* sample, rapid DNA extraction was performed in droplet format. Cell lysis solution was added, mixed, and heated in the 80°C PCR thermocycler chamber for 5 min. The lysed sample solution was redeposited onto the superhydrophobic surface and allowed to cool. 70% isopropanol solution was added to precipitate the DNA. After an initial mixing, the pipet tip rotated slowly to allow precipitated DNA to adhere to the tip. The tip was then removed and air dried for 1 min, washed in 70% ethanol, and air dried again for 1 min. Finally, the tip was mixed in DNA rehydration solution for 3 mins. Primers, Taq polymerase, and RNase free water were then added, mixed, and extracted for rapid-PCR thermocycling.

### Rapid droplet thermocycling

PCR was first performed under a conventional PCR machine to ensure proper design of primers and to serve as positive controls. PCR was run according to what was recommended by the GoTaq Green Master Mix (catalog number M7122; Promega Bio-Tek, Madison, WI, USA). Once positive results were verified, we employed our wire-guided droplet PCR system to detect the presence of *E. coli*.

AccessQuick® PCR system kit was used for the wire-guided droplet PCR reaction. A cocktail mixture which includes 4 μL of GoTaq Green, 1 μL of each 10 μM forward and reverse primers, 1 μL of DNA sample, and 3 μL of autoclaved water, for a total of 10 μL droplets, were used to run 30 PCR cycles. Following DNA extraction, the system automatically secured a 14-gauge blunt-ended syringe needle (modified for friction fit) and pulled-in the PCR-ready solution (DNA solution + Taq solution + primers). The hanging pendant droplet began the cycles in the 105°C chamber for denaturing, 55°C chamber for annealing of primers, and then 80°C chamber for extension of the products (See the Results and discussion section for the actual temperature of a droplet with these set temperatures). The final step consisted of final annealing for 20 s. During each of these stages, the system would move the hanging pendant droplet in a circular motion to aid in forced convective heat transfer. The chambers were connected with narrow bridges to allow the syringe needle to stay submerged in the silicone oil (catalog number S159-500; Fisher Scientific, Pittsburgh, PA, USA), preventing the possibility of evaporation or droplet loss due to the effects of surface tension from inserting and removing the needle from the oil.

### Primer design

Nucleotide sequences of aminoacyl-histidine dipeptidase (pepD) from *Escherichia coli* were pooled from GenBank [13]. These sequences were then subjected to multiple alignment analysis using ClustalX [14] to search for a conserved region so that consensus primers could be designed. Selected primers were then analyzed for appropriate melting temperature as well as any possible hairpin or self-dimerization by using OligoAnalyzer 3.1 (IDT Corporation, Coralville, IA, USA). Primers for pepD are 4F (5’- GGG AAT TCG TCG ACG TGT CTG AAC TGT CTC AAT T-3’) and 4R (5’- GAG CCG AAG CTT TTA CTT CGC CGG AAT TTC TT-3’) which results in about 1500 base pairs.

### Conventional DNA extraction of *E. coli*

DNA was extracted from *E. coli* that was cultured in Luria-Bertani (LB) media (catalog number MBPE-1050; Growcells, Irvine, CA, USA) overnight at room temperature to prevent cells from reaching death phase using Wizard Genomic DNA purification kit (catalog number A1120; Promega Bio-tek, Madison, WI, USA). Basically, cells were collected either by conventional centrifugation or centrifugation from the droplet manipulation system; and then subjected to the cell lysis solution to lyse cells and RNase solution to deactivate nucleases from breaking DNA and RNA. After an incubation period, DNA was precipitated with isopropanol and collected using centrifugation or the droplet manipulator. Samples were then washed with 70% ethanol and allowed to dry at room temperature for 15 min before rehydrate with rehydration solution. DNA was then subjected to conventional PCR for positive controls and rapid-PCR using droplet manipulations.

### Gel electrophoresis for PCR analysis

PCR product was determined through gel electrophoresis and fluorescent imaging of gel. PCR products were applied into 2% low melting agarose gel (catalog number E-3126-25; ISC BioExpress, Kaysville, UT, USA), using a Fisher Scientific (catalog number FB200) power supply at 80 V and 1 A for 60 min in a 1x tris-acetate-EDTA (TAE) buffer solution (catalog number BP13324; Fischer Scientific). The gel was soaked in about 1 μg mL^-1^ ethidium bromide for ca. 6 min and imaged using a Gel Doc 1000 imaging system (Bio-Rad Laboratories, Hercules, CA, USA).

### FLUENT modeling for droplet centrifuging

In order to track *E. coli* particles inside the droplet and to estimate the appropriate time for droplet centrifugation prior to DNA extraction, the commercial software (FLUENT 6.3 and GAMBIT 1.3; Fluent Inc., Lebanon, NH, USA) was used to build mathematical grids for our droplet PCR system using the finite volume method (FVM) [15]. A 2-D model was used in this study rather than a 3-D model as our system was symmetric in shape and it was assumed that the *E. coli* particles were generated and migratory at the face parallel to the direction of gravity due to centrifugation and perpendicular to the axis of rotation.

The grid size used in the simulation consisted of 70642 cells, 106277 faces and 35636 nodes. The shape of all meshes was triangular. First-order schemes were used as it is known to provide better convergence of calculations than the second-order, although it provides less accurate results due to the increased error in numerical discretization [16]. An unsteady-state solution was used in order to track the movement of the *E. coli* particles by time. Since the force from centrifugation was the dominant force of interest inside the droplet, neither the energy equation nor turbulent model options were chosen in FLUENT. Instead, a discrete phase model was used to track the *E. coli* particles. It was assumed that size of the droplet was 4 mm in diameter. The boundary was divided evenly into four zones by distance from the bottom of the droplet (zone 1: 2–3 mm from the bottom; zone 2: 1–2 mm from the bottom; zone 3: 0–1 mm from the bottom). Different gravitational forces were applied to each zone, to mimic the force from centrifugation as a function of the radius of the droplet region from the needle axis (zone 1: 58.86 m·s^-2^; zone2: 107.91 m·s^-2^; zone 3: 215.82 m·s^-2^). For the migratory simulation, it was assumed that the *E. coli* particles started from the top of zone 1 and migrated towards the bottom. This top to bottom migration represents the movement of the *E. coli* from the inside to the outside of the droplet. Five cases of simulations were conducted by changing the size of *E. coli* particle (diameter of 1, 2, 5, 7 and 10 μm) to simulate colony formation. The *E. coli* particles were assumed to be a solid sphere with density of 1.2 g cm^-3^. The time required for each *E. coli* colony to migrate to the bottom of the droplet was estimated for each case.

## Competing interests

The authors declare that they have no competing interests.

## Authors’ contributions

DJY and JYY conceived the original idea, analyzed the experimental and simulation results and wrote the manuscript jointly. DJY designed/fabricated the apparatus, and performed all experiments. Both authors read and approved the final manuscript.

## Supplementary Material

**A complete movie showing the wire-guided droplet manipulations for serial dilution, droplet centrifugation, droplet DNA extraction, and rapid droplet thermocycling.** Click here for file
